# Type III interferon-induced CBFβ inhibits HBV replication by hijacking HBx

**DOI:** 10.1038/s41423-018-0006-2

**Published:** 2018-03-09

**Authors:** Fengchao Xu, Hongxiao Song, Qingfei Xiao, Na Li, Hong Zhang, Genhong Cheng, Guangyun Tan

**Affiliations:** 1 Department of Immunology, Institute of Translational Medicine, The First Hospital of Jilin University, Changchun, Jilin 130061 China; 2 Department of Nephrology, The First Hospital, Jilin University, Changchun, Jilin 130021 China; 3 Department of Obstetrics, The First Hospital of Jilin University, Changchun, Jilin 130021 China; 4 Phase I Clinical Research Center, The First Hospital of Jilin University, Jilin, 130021 China; 5 Department of Microbiology, Immunology and Molecular Genetics, University of California, Los Angeles, CA 90095 USA; 6 Center of Systems Medicine, Institute of Basic Medical Sciences, Chinese Academy of Medical Sciences and Peking Union Medical College, Beijing 100005; Suzhou Institute of Systems Medicine, Suzhou, Jiangsu 215123 China

## Abstract

Hepatitis B virus (HBV) and its associated chronic infection remain serious health threats worldwide. However, there is still no impactful approach for clinical treatment of hepatitis B patients. Therefore, developing a better understanding of the interactions between HBV and its host is particularly important. HBV infection has been reported to induce type-III but not type-I or type-II interferon (IFN). In this study, we identified CBFβ, an HIV enhancer, as an HBV restriction factor that is specifically induced by type-III IFN in the early stages of HBV infection. Type-III IFN-induced IL-10 played an important role in the production of CBFβ. Interestingly, the interaction between CBFβ- and HBV-encoded regulatory protein X (HBx) enhanced the stability of CBFβ, but notably blocked HBx-mediated promotion of HBV replication. CBFβ expression was lower in HBV patients than in healthy persons, and the addition of serum from HBV patients inhibited CBFβ expression in HepG2 cells. On the contrary, HBV via HBsAg inhibited type-III IFN-induced CBFβ expression and decreased the anti-HBV activity of type-III IFN, suggesting that HBV inhibits antiviral interferon-stimulated gene (ISG) expression and induces IFN resistance. Collectively, our results demonstrate that type-III IFN-triggered and IL-10-induced CBFβ are crucial factors for inhibiting HBV replication, and the HBx–CBFβ–HBsAg axis reveals a new molecular mechanism of interaction between HBV and its hosts.

## Introduction

Three-quarters of the world’s population live in areas where there are high levels of hepatitis B virus (HBV) infection. Despite the use of HBV vaccination for almost 30 years, 240 million people out of 2 billion HBV-infected people worldwide are chronically infected with HBV and remain at high risk of developing liver cirrhosis and hepatocellular carcinoma (HCC).^1,2^ According to the study on the Global Burden of Disease (2010), HBV infection is the tenth leading cause of death, and more than 780,000 patients die each year due to HBV-related diseases.^3^ HBV is a partially double-stranded DNA virus that belongs to the *Hepadnaviridae* family. All HBV viral RNAs are transcribed from HBV covalently closed circular DNA (cccDNA). Following HBV entry into host cells, cccDNA is converted to RNA in the nucleus and acts as an origin for HBV-persistent infections.^4^

The HBV-encoded regulatory protein X (HBx) is a 17-kDa protein that consists of an N-terminal negative regulatory domain and C-terminal transactivation or coactivation domain.^5^ HBx interacts with several cellular proteins and may mediate its role in virus replication through these interactions. Damage-specific DNA-binding protein 1 (DDB1) is the best-characterized HBx-binding partner,^6^ and the interaction of HBx and DDB1 is essential for HBV replication. Several reports have shown that the HBx–DDB1–CUL4–ROC1 E3 ligase complex targets the SMC5/6 complex to enhance HBV gene expression from episomal cccDNA.^7,8^

Interferons (IFNs) are a group of signaling proteins made and released by host cells in response to several pathogens, such as bacteria, viruses, or parasites.^9,10^ Based on the type of receptor through which they signal, human interferons are classified into three major types: type-I, type-II, and type-III.^11^ Type-I interferon (IFN-α, IFN-β, IFN-ε, IFN-κ, and IFN-ω) is currently approved for the treatment of chronic HBV,^9^ and IFN-α has been reported to inhibit HBV replication in other systems in vitro.^12,13^ However, these treatment strategies are associated with poor response rates and substantial side effects, which impact their clinical utility. HBV has been found to inhibit IFN-stimulated genes (ISGs) and type-I IFN in infected patients.^14–17^ Type-III IFN (IFN-λs; IL-29/IFN-λ1, IL-28A/IFN-λ2, and IL-28B/IFN-λ3) binds to IL-28 receptor α (IL-28Rα), while IL-10Rβ is a potential partner subunit for IL-28Rα.^18^ It has been reported that HBV infection induces type-III IFN but not type-I or type-II IFNs^19^ and also inhibits HBV replication.^20,21^ Therefore, type-III IFN might play an indispensable role in anti-HBV processes after HBV infection. However, the molecular mechanism remains to be fully clarified.

Core-binding factor subunit β (CBFβ) is a protein that increases the steady-state level of viral infectivity factor (Vif) protein by promoting APOBEC3G polyubiquitination and degradation, which in turn facilitates HIV replication.^22,23^ CBFβ binds with Runt-related transcription factors 1, 2, and 3 (RUNX1, 2, and 3) and acts as a critical regulator in hematopoiesis, T-cell differentiation, and bone development.^24,25^ However, to the best of our knowledge, there are few reports that have discussed CBFβ in terms of regulating HBV and CBFβ expression. Here, we found that type-III IFN-triggered and IL-10-induced CBFβ inhibited HBV replication. The interaction of HBx and CBFβ enabled a more stable CBFβ, which could enhance the inhibition of HBV replication by CBFβ. In addition, hepatitis B virus surface antigen (HBsAg) might be responsible for the low expression of CBFβ in HBV patients.

## Methods

### Cell culture, plasmids, and reagents

HEK293T, HepG2.2.15, and HepG2 cells were maintained in DMEM medium containing 10% inactivated fetal bovine serum. HLCZ01 cells were maintained according to the previous report.^26^ All cell lines were maintained in penicillin (100 IU/mL) and streptomycin (100 mg/mL) in the presence of 5% CO_2_ at 37°C. The expression construct of CBFβ was generated by cloning the coding region sequence into the VR1012 expression vector, and CBFβ mutants were generated by Quik-Change PCR (TransGen, Beijing, China) (Table S1). The pHBV1.3 plasmid was kindly supplied by Dr. Lishan Su at the University of North Carolina, and the pHBV1.2 WT and ΔX plasmids were kindly provided by Professor Qiang Deng of Fudan University. Antibodies against tubulin were obtained from Santa Cruz Biotechnology (Santa Cruz, CA). Antibodies against CBFβ were from Cell Signaling Technology (Danvers, MA). Antibodies against GAPDH were obtained from Proteintech. IFIT2 antibodies were obtained from Signalway Antibody. Smc6 antibodies were purchased from Abcam (Shanghai, China). Human IL-10, IL-29 (IFN-λ1) recombinant protein, and IL-10 and IL-29 neutralized antibodies were obtained from R&D SYSTEMS (Minneapolis, MN, USA). Human IL-10 and IL-29 (IFN-λ1) ELISA Kits were purchased from ORIGENE (USA).

### RNA extraction and quantitative real-time PCR (Q-PCR)

Total RNA was extracted from cells using EasyPure RNA Kit (Transgen, China) according to the manufacturer’s instructions and then was converted to first-strand cDNA using TransScript First-Strand cDNA Synthesis SuperMix (Transgen, China). HBV DNA was isolated from supernatants according to the kit instructions (Transgen, China). The housekeeping gene GAPDH was used as an internal control for quantitation, and gene expression was quantified as previously described.^27^ The gene-specific primer sequences used for Q-PCR are shown in Table S1.

### Co-immunoprecipitation and Western blot analysis

At 24–48 h after transfection of the expression plasmids, cells were lysed with 50 mM Tris–HCl, pH 8.0, 150 mM NaCl, and 1% NP-40 containing cocktail inhibitors (Roche). Cell lysates were immunoprecipitated and incubated with ANTI-FLAG® M2 Affinity Gel (Sigma) overnight. Immunoblotting was carried out as described previously.^28^ Briefly, cells were collected and lysed in ice-cold cell lysis buffer (20 mM HEPES, 350 mM NaCl, 20% glycerol, 1% NP-40, 1 mM MgCl_2_, 0.5 mM EDTA, 0.1 mM EGTA, and 0.5 mM DTT) for 30 min and by tapping the tubes every 10 min. The protein concentrations of the lysates were quantified by Coomassie Plus protein assay Reagent (Thermo Scientific). Quantification of the band intensities was carried out with ChemiDoc XRS^+^ Molecular Imager software (Bio-Rad). Samples were separated by SDS-PAGE and transferred onto PVDF membranes. The blots were blocked in TBS containing 0.1% Tween-20 and 5% skimmed milk and were then probed with the relevant antibodies.

### Enzyme-linked immunosorbent assay (ELISA)

HepG2 cells were mock transfected or transfected with the CBFβ-expression plasmid and pHBV1.3-HBV expression plasmid. Supernatants were collected after 72 h, and the supernatants from HLCZ-01 cells were collected on the 7th day after infection for analysis by ELISA to detect the levels of HBeAg and HBsAg (Kehua Shengwu, China).

### CRISPR/Cas9 knockout

HepG2 cells were seeded in 24-well plates, and 16 h later, plasmids expressing Cas9, CBFβ sgRNA individually, and a plasmid containing a puromycin selection maker were cotransfected into HepG2 cells using Viafect transfection reagent (Promega). At 36 h post transfection, cells were either selected by adding puromycin at a concentration of 2 µg/ml or subjected to immunoblotting with CBFβ-specific antibodies (Cell Signaling). Two days later, serial dilution of living cells was performed to obtain one cell per well in a 96-well plate. Immunoblotting was performed to ensure gene knockout (KO) after the clones grew, and DNA sequencing was performed to further confirm gene KO. The sgRNA sequences are shown in Table S1.

### HBV infection assay

The supernatants of HepG2.2.15 cells, which were derived from the human hepatoma cell line HepG2 transfected with the full genome of HBV, were collected and concentrated. HLCZ01 cells were seeded into 60-mm plates, and 16 h later, cells were transfected with 3 µg of HA-CBFβ plasmids or control plasmids and with 150 ng of green fluorescent protein (GFP) expression plasmids for each transfection; 36 h later, GFP-positive cells were sorted into 24-well plates and cultured overnight. Then, cells were inoculated for 2 days with a multiplicity of infection (MOI) of 100 genome equivalents (Geq) per cell; after infection, cells were washed three times with PBS and were maintained in DMEM/F12 medium for another 7 days. The supernatants and cells were collected for HBV DNA, pgRNA, HBsAg, and HBeAg detection by Q-PCR or ELISA.

### Samples

Twenty-eight HBV-infected patients, 15 healthy people, and 10 HBV patients who were receiving IFN-α treatment were enrolled in this study (Table S2, S3). Venous blood was withdrawn to collect serum and PBMCs. These studies were approved by the IRB of Jilin University, the First Hospital.

### Statistical analysis

The results are presented as the mean ± s.d. and were analyzed using Student's *t*-test. *P* < 0.05 was considered statistically significant.

## Results

### CBFβ is upregulated in the early stages of HBV infection

**Fig. 1:**
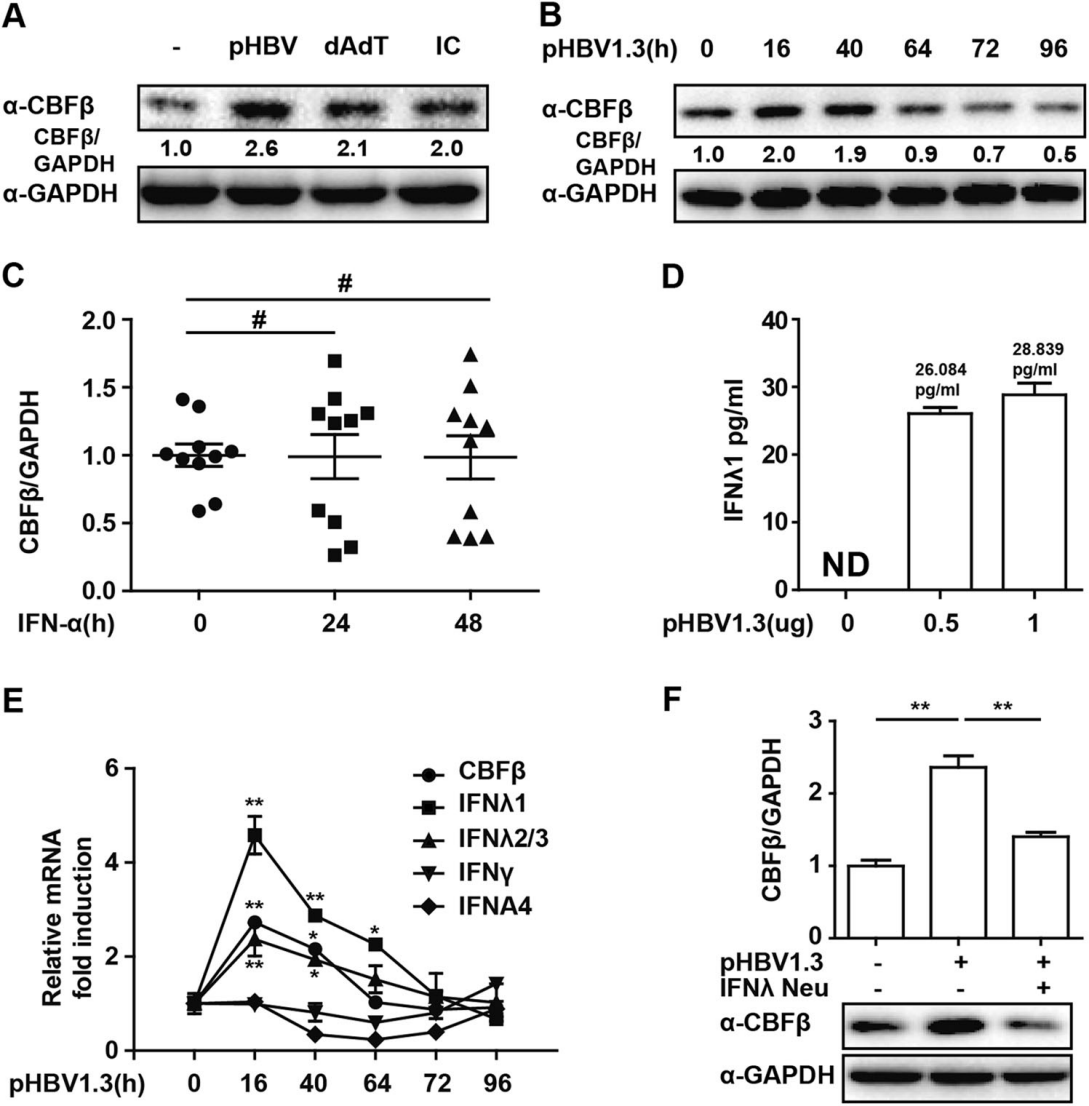
CBFβ induction in the early stage of HBV infection. **a** HepG2 cells were transfected with pHBV1.3 plasmids, polydAdT or polyIC, as indicated; 16 h later, whole-cell lysates were immunoblotted with CBFβ or GAPDH antibodies. **b** HepG2 cells were transfected with pHBV1.3 plasmids, and cells were collected at different time points as indicated; whole-cell lysates were subjected to immunoblotting as in **a**. **c** PBMCs from HBV patients before or after IFN treatment were isolated and subjected to RNA extraction, and Q-PCR was performed to evaluate the CBFβ mRNA level. **d** HepG2 cells were transfected with pHBV1.3 plasmids as indicated. Then, 72 h later, supernatants were collected, and IFN-λ1 was analyzed by ELISA. **e** HepG2 cells were transfected as in **b**. Cells were collected and subjected to RNA extraction and Q-PCR analysis to measure CBFβ, IFN-λ1/2/3, IFN-γ, and IFNA4 expression. **f** HepG2 cells were transfected with pHBV1.3 plasmids and treated with IFN-λ1-neutralizing antibody as indicated; a western blot and Q-PCR were performed to evaluate the CBFβ level. All of the data are from three pooled independent experiments and are shown as the mean ± s.d. *p < 0.05, **p < 0.01, ^#^*p* > 0.05

CBFβ has been reported to be hijacked by HIV Vif protein to degrade APOBEC3G and promote HIV infection.^22^ To study the regulation of CBFβ in response to HBV infection, with polydAdT and polyIC as controls, we found that CBFβ was significantly upregulated in HepG2 cells transfected with the HBV whole-genome expression plasmid (pHBV1.3; Fig. 1a) and that it was downregulated in a time-dependent manner (Fig. 1b). Activation of IFN is the earliest transcriptional response to viral infection, and the major antiviral effect of IFN is achieved by induction of ~300 IFN-inducible genes, which results in the direct or indirect inhibition of the virus.^29^ However, we found that CBFβ could not be induced after IFN-α treatment in HBV patients (Fig. 1c). Consistent with previous reports, type-III IFN was induced after pHBV1.3 transfection in HepG2 cells (Figs. 1d, e), and we presumed that CBFβ was induced by type-III IFN. As expected, the induction of CBFβ was significantly correlated with IFN-λ kinetics in response to HBV replication in HepG2 cells (Fig. 1e). In addition, HBV-induced CBFβ was inhibited at both the protein and mRNA levels by treating cells with an IFN-λ-neutralizing antibody (Fig. 1f). Collectively, these results suggested that CBFβ was induced after HBV infection and that CBFβ might act as a type-III IFN-inducible gene.

### CBFβ is a type-III IFN-inducible gene

**Fig. 2:**
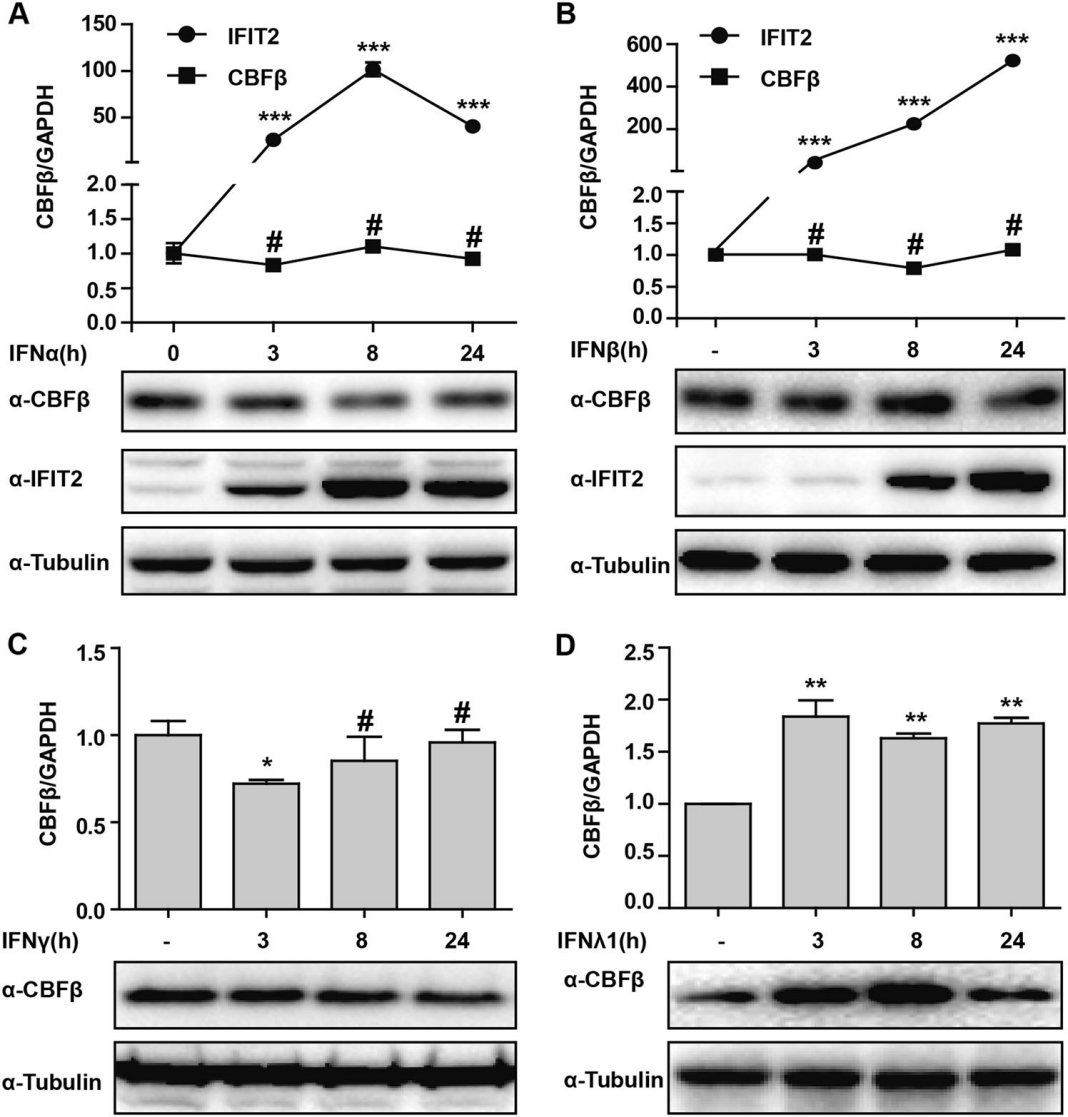
CBFβ is specifically induced by IFN-λ. **a, b** HepG2 cells were treated with IFN-α or IFN-β as indicated. Whole-cell lysates were immunoblotted with CBFβ, IFIT2, or GAPDH antibodies, and RNA was extracted and subjected to Q-PCR analysis to measure the CBFβ or IFIT2 expression. **c** HepG2 cells were treated with IFN-γ as in **a**, **b**, and analyzed as in **a**, **b**. **d** HepG2 cells were treated with IFN-λ1 as in **a**, **b**, and analyzed as in **a**,** b**. All of the data are from three pooled independent experiments and are shown as the mean ± s.d. Student’s *t-*test was performed. *p < 0.05, **p < 0.01, ^#^*p* > 0.05

To confirm our speculation that CBFβ is induced by type-III IFN, we further treated HepG2 cells with IFN-α/β, IFN-γ, or IFN-λ1/3 as indicated. Cells were then collected and analyzed by using Q-PCR and western-blotting analysis. The results revealed that tetratricopeptide repeats 2 (IFIT2), a type-I IFN-inducible gene, was induced by IFN-α or IFN-β treatment, while HepG2 cells treated with IFN-α and IFN-γ showed no induction of CBFβ in both mRNA and protein levels over time (Fig. 2a–c). However, both the protein and mRNA levels were significantly increased after IFN-λ1 or IFN-λ3 stimulation (Fig. 2d and Fig. S1). Taken together, these results suggested that CBFβ was a type-III IFN-inducible gene and might be induced by type-III IFN after HBV infection.

### **IL-10 plays a pivotal role in CBFβ induction by type-III IFN**

**Fig. 3:**
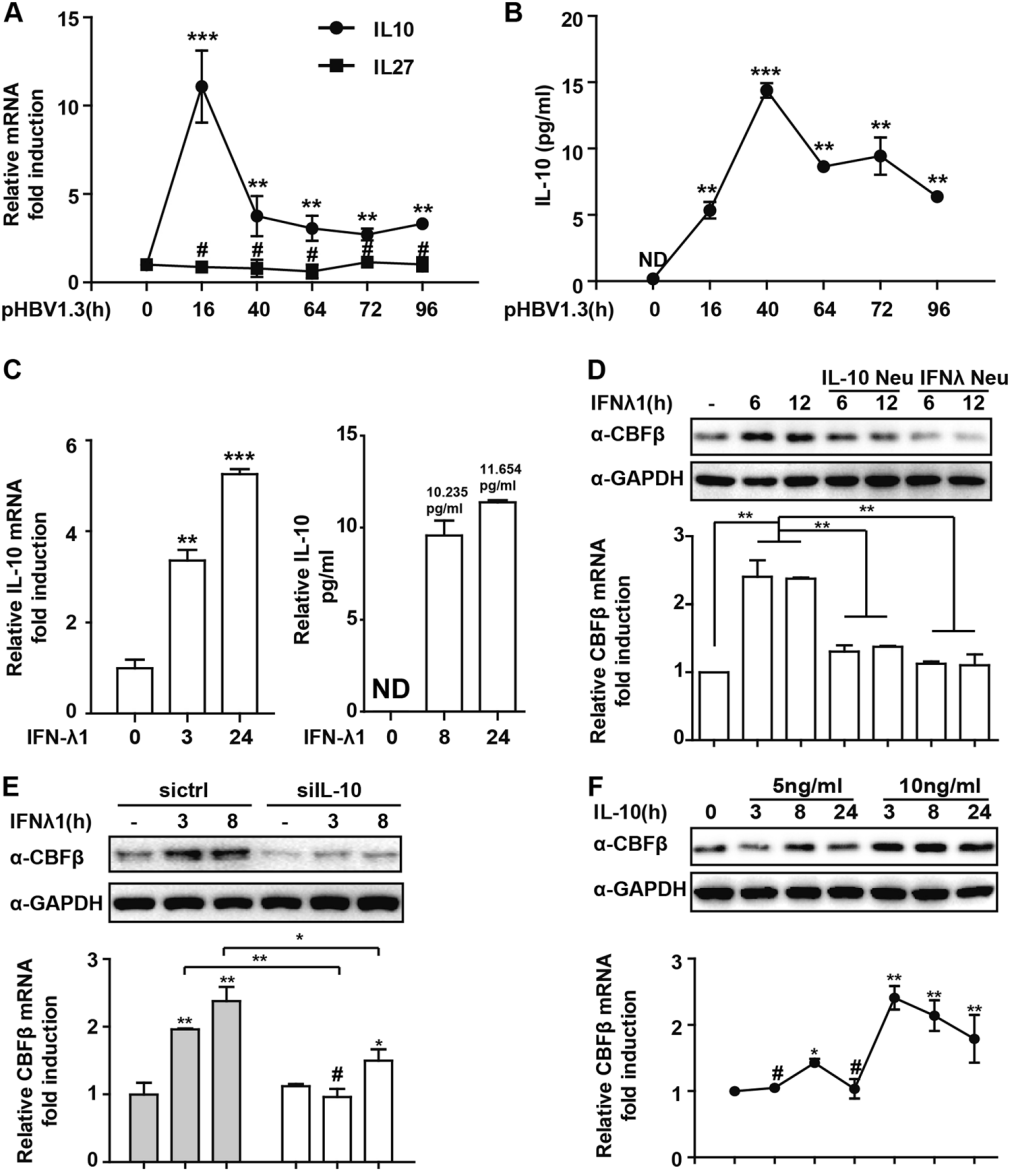
Type-III IFN-induced CBFβ production is IL-10 dependent. **a** HepG2 cells were transfected with pHBV1.3 or left untransfected as indicated; RNA was extracted and subjected to Q-PCR analysis to measure IL-10 or IL-27. **b** The supernatant from A was evaluated to detect the IL-10 level by ELISA. **c** HepG2 cells were treated with IFN-λ1 as indicated; Q-PCR or ELISA was performed to analyze the IL-10 mRNA or protein level. **d** HepG2 cells were treated with IFN-λ1 or together with IFN-λ1 or IL-10-neutralizing antibody as indicated, whole-cell lysates were immunoblotted with CBFβ or GAPDH antibodies, and RNA was extracted and subjected to Q-PCR analysis to measure CBFβ expression. **e** HepG2 cells were transfected with siRNA control or siIL-10 as indicated and analyzed as in **d**. **f** HepG2 cells were treated with IL-10 protein as indicated and analyzed as in **d**. All of the data are from three pooled independent experiments and are shown as the mean ± s.d. *p < 0.05,**p < 0.01, ***p < 0.001, ^#^*p* > 0.05

Next, we investigated how type-III IFN induced CBFβ. We previously studied the regulatory functions of IL-10 and IL-27^28,30^ and evaluated whether IL-10 or IL-27 was required for type-III IFN to induce CBFβ. Interestingly, IL-10 but not IL-27 was induced in pHBV1.3-transfected HepG2 cells (Fig. 3a, b), showing similar kinetics with CBFβ and IFN-λ induction (Fig. 1e). We also found that IL-10 was significantly upregulated in response to IFN-λ stimulation in HepG2 cells (Fig. 3c). Therefore, HepG2 cells were treated with an IL-10-neutralizing antibody, which blocked the induction of CBFβ by IFN-λ (Fig. 3d). IL-10 knockdown significantly inhibited IFN-λ-mediated CBFβ induction (Fig. 3e and Fig. S2A). HepG2 cells were further treated with IL-10 recombinant proteins, and we found that the CBFβ mRNA and protein levels were significantly upregulated (Fig. 3f). Interestingly, IL-10 was not induced by either IFN-α or IFN-γ in HepG2 cells, and this low expression of IL-10 might explain why IFN-α or IFN-γ could not induce CBFβ (Figure S2B). Collectively, these results indicated a pivotal role of IL-10 in CBFβ induction by type-III IFN.

### CBFβ inhibits HBV replication

**Fig. 4:**
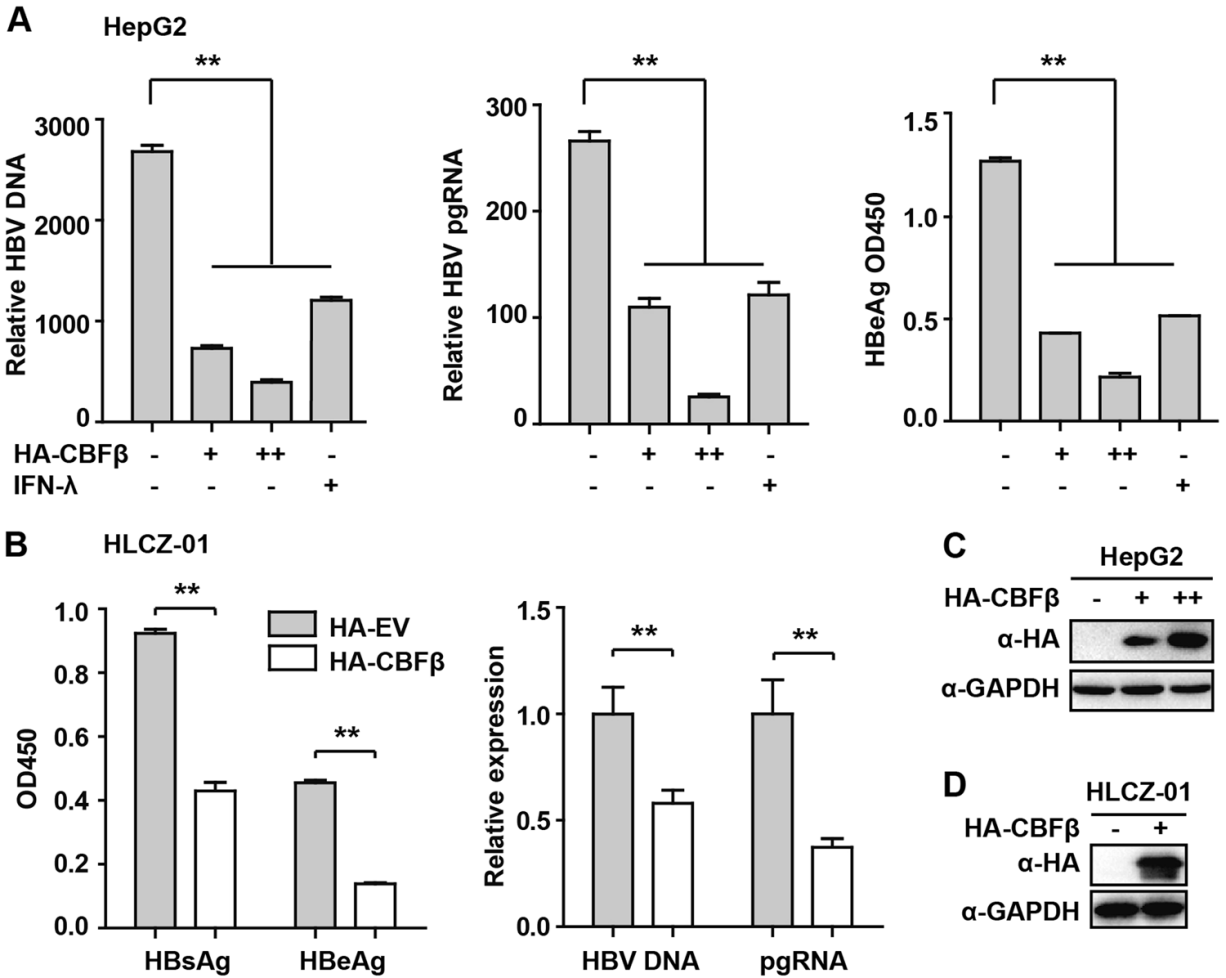
CBFβ inhibits HBV replication. **a**, **c** HepG2 cells were pretreated with IFN-λ1 for 8 h as a positive control or left untreated; after treatment, cells were transfected with pHBV1.3 or cotransfected with pHBV 1.3 and with different amounts of CBFβ as indicated. At 72 h, the supernatant was collected, and cells were subjected to immunoblotting with antibodies as indicated. RNA and HBV DNA were isolated from cells or supernatants and analyzed by Q-PCR. HBeAg in the supernatant was analyzed by ELISA. **b, d** HLCZ-01 cells were transfected with CBFβ or empty vector together with GFP plasmids; 36 h later, GFP-expressed cells were sorted to 24-well plates and infected with HBV. Then, 7 days after infection, the cells and supernatants were collected and analyzed by Q-PCR or ELISA, and the cells were subjected to immunoblotting with antibodies as indicated. All of the data are from three pooled independent experiments and are shown as the mean ± s.d. *p < 0.05, **p < 0.01

Following the above results, we further investigated the function of CBFβ in HBV replication. The pHBV1.3 plasmid can be used to transfect HepG2 cells and create infectious HBV virus. This plasmid was cotransfected with HA-tagged CBFβ-expressing plasmids to evaluate the possible effects of CBFβ on viral replication. Cells and supernatants were collected at 72 h after transfection. ELISA and Q-PCR were performed to quantify the protein levels of hepatitis B e antigen (HBeAg), HBV DNA, and HBV pregenomic RNA (pgRNA). Notably, similar results were obtained as those observed with IFN-λ treatment, where HBV DNA, pgRNA, and HBeAg were significantly reduced after CBFβ overexpression (Fig. 4a, c). In our investigation to further verify the role of CBFβ in HBV infection, HLCZ01, a liver cell line that has been reported to support the entire life cycle of both HBV and HCV, was used in our HBV infection assay.^26^ Consistently, HBV DNA, pgRNA, HBsAg, and HBeAg were significantly reduced in CBFβ- overexpressed HLCZ01 cells (Figs. 4b, d). These data suggested that CBFβ played an inhibitory role in regulating HBV replication.

### CBFβ is essential for IFN-λ-mediated inhibition of HBV

**Fig. 5:**
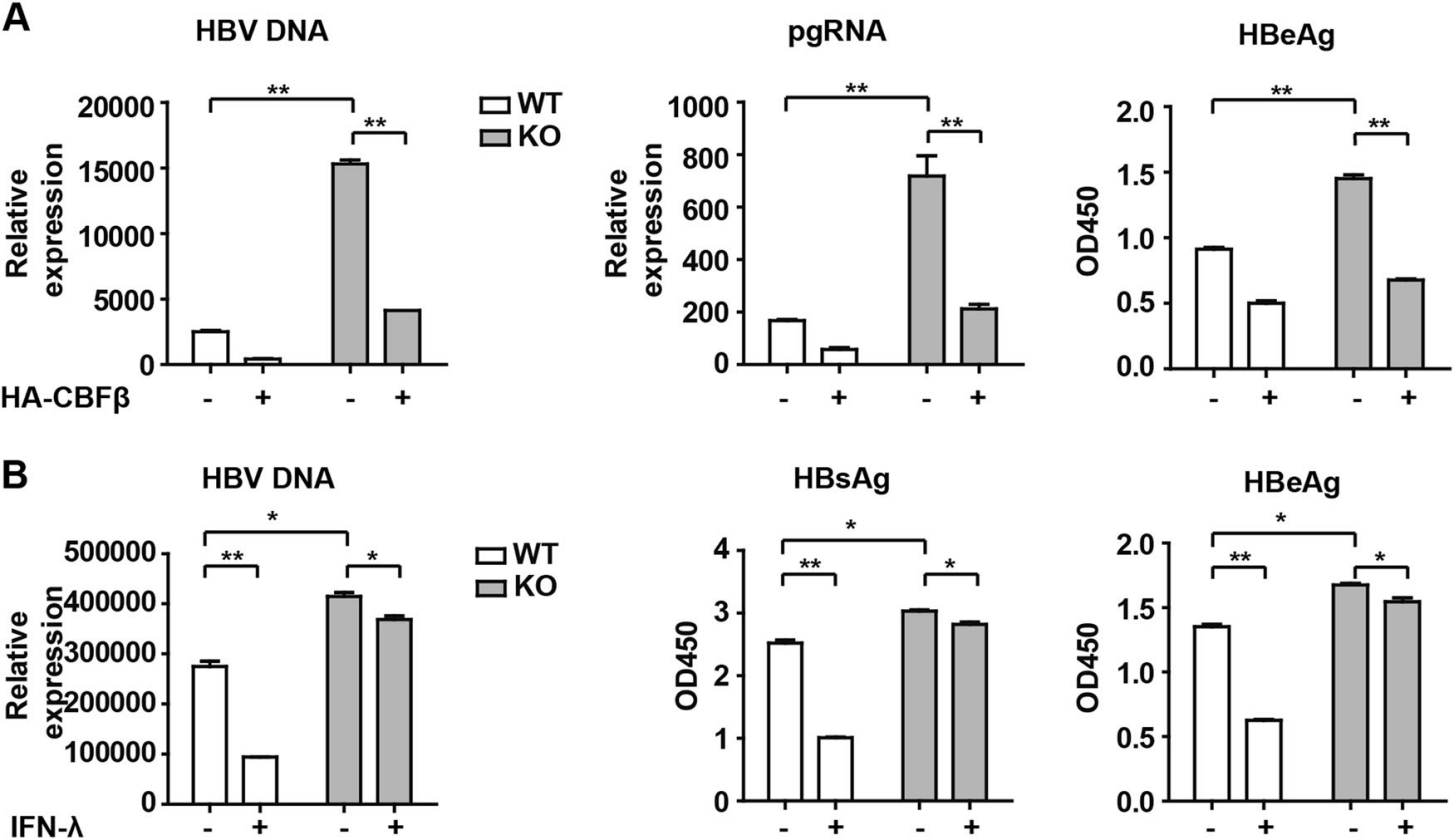
CBFβ is essential for IFN-λ-mediated inhibition of HBV. **a** HepG2 WT and CBFβ KO cells were transfected with pHBV1.3 or cotransfected with pHBV 1.3 and CBFβ. Cells and supernatants were collected at 72 h; HBV DNA was isolated from the supernatants and analyzed by Q-PCR. HBeAg in the supernatant was analyzed by ELISA. Data are presented as the means and s.d. from three independent experiments. Student’s *t*-test was performed. **p < 0.01. **b** HepG2 WT and CBFβ KO cells were pretreated with IFN-λ1 for 8 h and transfected with pHBV1.3; 48 h later, the cells and supernatants were collected and analyzed as in **a**. All of the data are from three pooled independent experiments and are shown as the mean ± s.d. *p < 0.05, **p < 0.01

To further confirm our results, CBFβ was knocked out (KO) using CRISPR/Cas9 technology in HepG2 cells. The results showed that HBV replication was enhanced in KO cells compared to WT cells and was restored upon reconstitution with CBFβ in KO cells using a HA-tagged plasmid expressing WT CBFβ (Fig. 5a and Fig. S3A). In addition, HepG2 WT or CBFβ KO cells were transfected with pHBV1.3 plasmids with or without IFN-λ treatment, and as expected, the inhibitory effects of IFN-λ on HBV replication were significantly attenuated in CBFβ KO cells (Fig. 5b and Fig. S3B). This result suggests an indispensable role of CBFβ in IFN-λ-mediated inhibition of HBV replication.

### CBFβ inhibits HBV replication by interacting with HBx

**Fig. 6:**
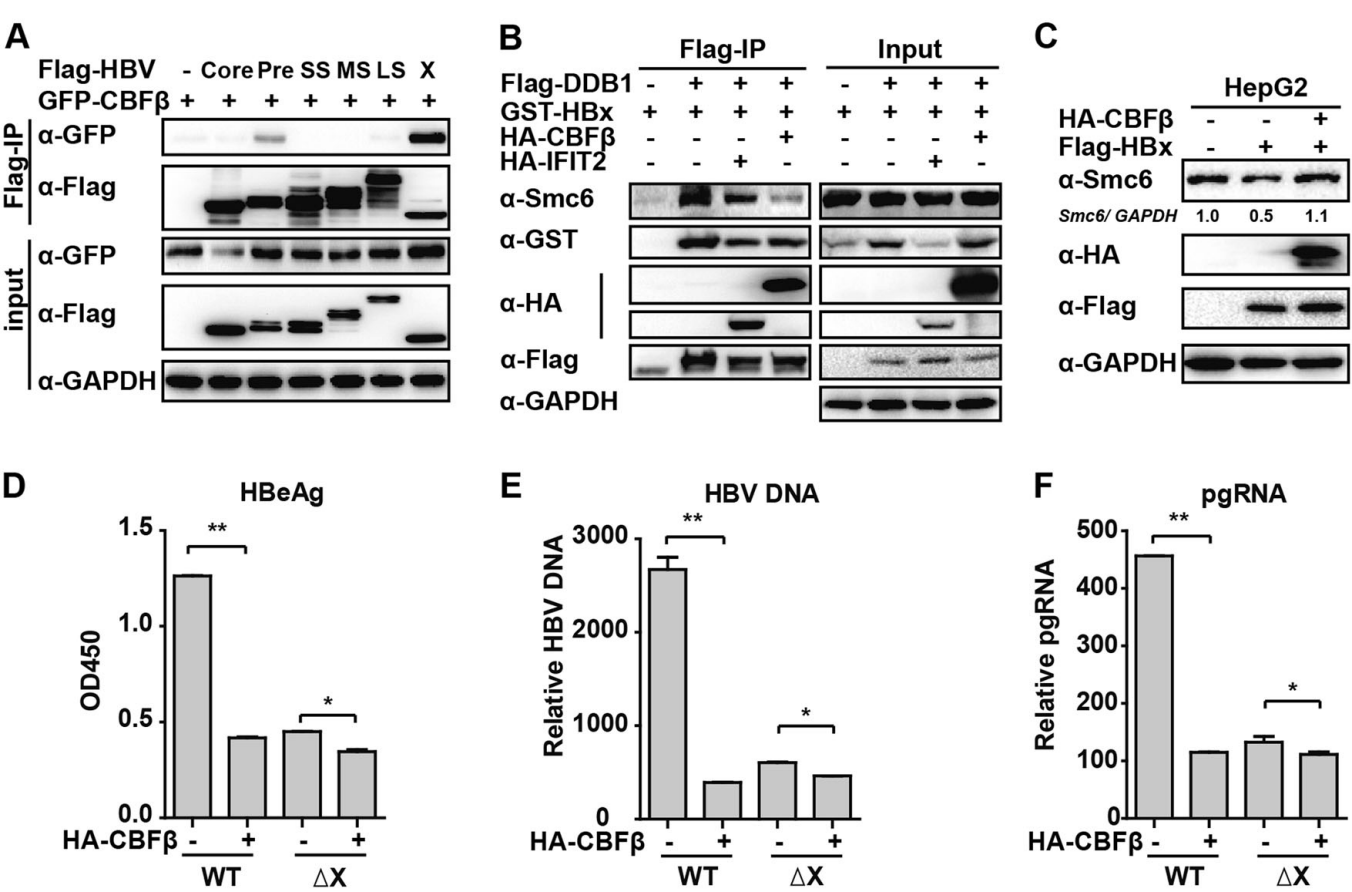
CBFβ inhibits HBV replication through interacting with HBx. **a** 293T cells were transfected with CBFβ or cotransfected with CBFβ and HBV protein expression plasmids as indicated; 24 h later, cells were collected and subjected to Co-IP assay, and the blot was immunoblotted with antibodies as indicated. **b** 293T cells were transfected with HA-CBFβ, HA-IFIT2, Flag-DDB1, or GST-HBx expression plasmids as indicated. Then, 32 h later, cells were collected and subjected to Co-IP assay, and the blot was immunoblotted with antibodies as indicated. (**c**) HepG2 cells were transfected with HA-CBFβ or Flag-HBx expression plasmids as indicated; 48 h later, cells were collected and subjected to immunoblotting with antibodies as indicated. **d–f** HepG2 cells were transfected with pHBV1.2 WT or pHBV 1.2ΔX plasmids, and with or without CBFβ as indicated. At 72 h, RNA and HBV DNA were isolated from the cells or supernatants and analyzed by Q-PCR. HBeAg in the supernatant was analyzed by ELISA. Data are presented as the means and s.d from three independent experiments. Student’s *t*-test was performed. *: *p* < 0.05, **: *p* < 0.01

Following the above results, we sought to verify how CBFβ inhibits HBV replication. Protein–protein interactions are perhaps the most direct form of the biological function of proteins.^31^ Based on this idea, we investigated whether CBFβ interacted with HBV proteins. Six HBV proteins, including HBV core, Pre-core, Small S, Middle S, Large S, and X, were cotransfected individually with the CBFβ expression plasmid into 293T cells. The results demonstrated that CBFβ interacted with HBx and Pre-core, but not with other HBV proteins (Fig. 6a), and that the interaction with HBx was much stronger than that of Pre-core. These results suggested that CBFβ might inhibit HBV replication by interacting with HBx and blocking HBx transcription activity. Smc5/6 forms the foundation of a multisubunit DNA repair complex and functions as a restriction factor by selectively blocking extrachromosomal DNA transcription.^32^ It has been reported that HBx binds the DDB1 subunit of the DDB1-containing E3 ubiquitin ligase to target Smc5/6 complexes for ubiquitin-mediated degradation.^33^ Therefore, we intended to determine whether CBFβ blocked HBx–DDB1–Smc complex formation and inhibited HBx-mediated degradation of Smc5/6; interestingly, as we expected, CBFβ but not IFIT2 (control) overexpression in 293T cells significantly inhibited the interaction of HBx, DDB1, and Smc6 (Fig. 6b), and CBFβ overexpression rescued HBx-mediated degradation of Smc6 (Fig. 6c). To further verify our conjecture, pHBV1.2 WT and ΔX (without HBx expression) were cotransfected into HepG2 cells; 72 h later, ELISA and Q-PCR were performed to quantify the protein levels of HBeAg, HBV DNA, and HBV pregenomic RNA (pgRNA). The results indicated that HBV replication was inhibited in pHBV1.2ΔX-transfected cells compared to pHBV1.2WT-transfected cells, suggesting the important role of HBx in promoting HBV replication. Interestingly, the function of CBFβ inhibition in HBV replication was much less significant in pHBV1.2ΔX-transfected HepG2 cells than in pHBV1.2 WT-transfected cells (Figs 6d–f), demonstrating that CBFβ inhibits HBV replication by targeting HBx. The loop3(69–90aa) and helix4 (129–140aa) regions of CBFβ were reported to be important for the interaction of CBFβ and HIV-1 Vif;^23^ we also tried to verify whether these two regions were indispensable in the interaction of CBFβ and HBx, but after deletion, the mutants still interacted with HBx and inhibited HBV replication (Figure S4), indicating that HBx interacted with other domains of CBFβ, which need to be further studied. Taken together, these results suggested that CBFβ might block HBx transcriptional activity through a protein–protein interaction and might inhibit the role of HBx in promoting HBV replication.

### HBx deubiquitinates CBFβ

**Fig. 7:**
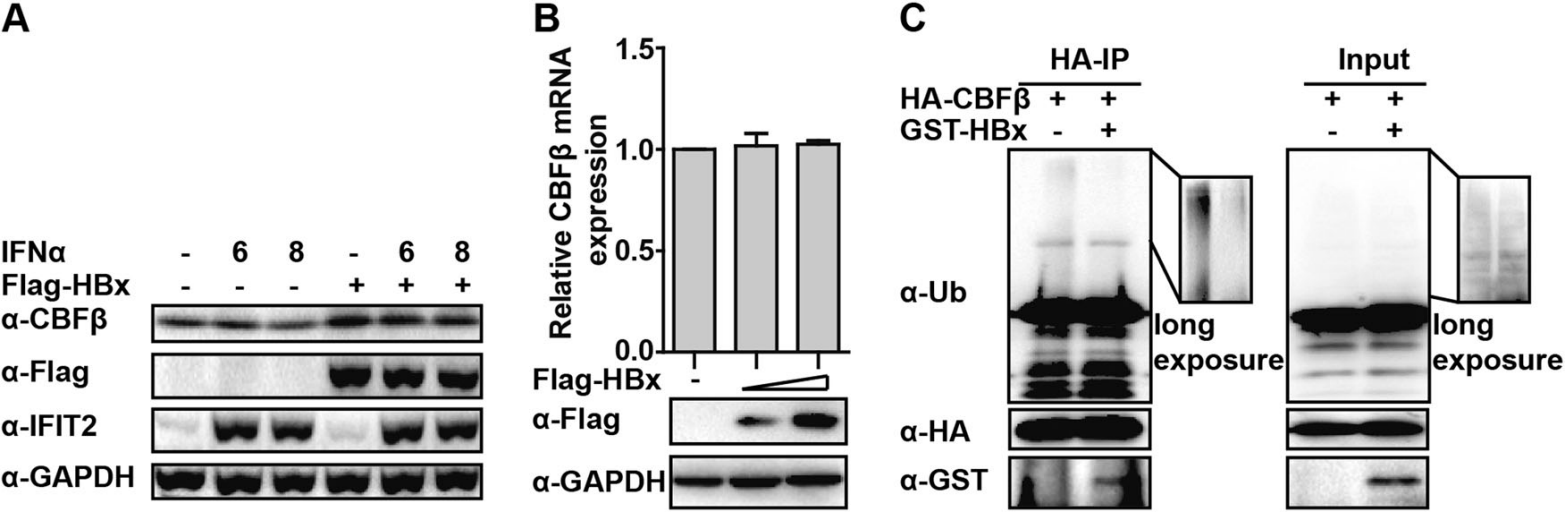
HBx enhanced CBFβ expression by deubiquitination. **a** HepG2 cells were transfected with Flag-HBx expression plasmids and treated with IFN-α as indicated; 36 h later, whole-cell lysates were prepared and immunoblotted with antibodies as indicated. **b** HepG2 cells were transfected with HBx expression plasmids; 36 h later, whole-cell lysates were subjected to immunoblotting using Flag and GAPDH antibodies, and Q-PCR was performed to analyze the CBFβ mRNA level. **c** HepG2 cells were transfected with CBFβ or cotransfected with CBFβ and HBx expression plasmids as indicated; 24 h later, cells were treated with MG132 for 8 h; then, whole-cell lysates were prepared and subjected to Co-IP assay, and the blot was immunoblotted with antibodies as indicated. All of the data are from three pooled independent experiments and are shown as the mean ± s.d. ^#^*p* > 0.05

In addition, we noted that CBFβ expression was higher in HBx-transfected samples (Fig. 6a, **input, line 7**), implying that the presence of HBx might increase CBFβ stability. This conjecture was confirmed by transfecting the HBx expression plasmid into HepG2 cells, which led to enhanced expression of CBFβ. Consistent with our results in Fig. 2, IFIT2 was upregulated by IFN-α, but did not induce CBFβ expression (Fig. 7a). Additionally, no effect on the expression of CBFβ mRNA by HBx was observed (Fig. 7b), which indicated that HBx might stabilize the CBFβ protein by promoting CBFβ deubiquitination. As expected, cotransfection with HBx significantly inhibited the ubiquitination of CBFβ in the co-immunoprecipitation (co-ip) assay (Fig. 7c). These results suggested that CBFβ might hijack HBx after HBV infection, which subsequently enhanced its stability and facilitated the inhibitory effect of CBFβ in HBV replication.

### HBsAg inhibits CBFβ expression

**Fig. 8:**
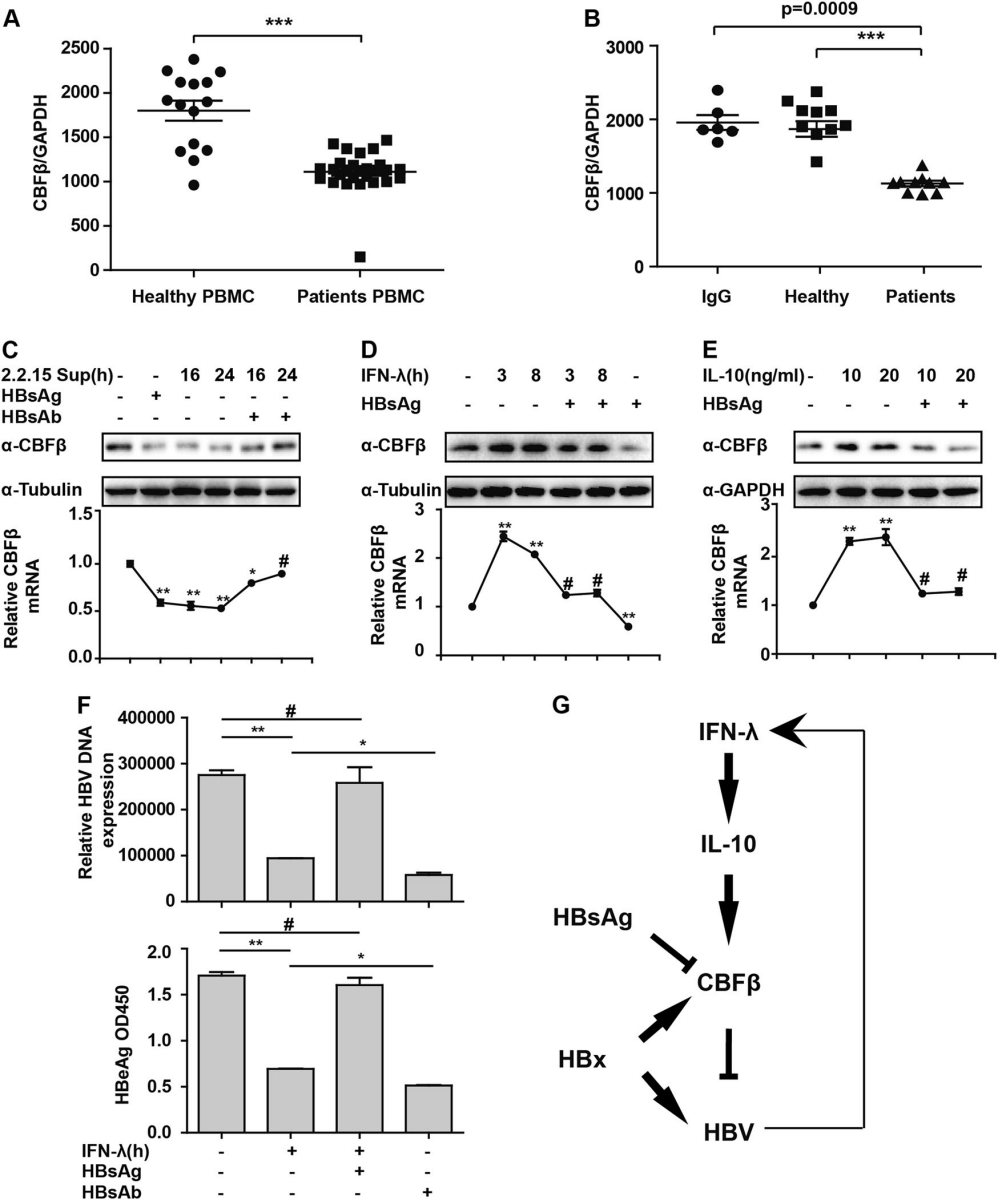
HBsAg inhibits CBFβ expression. **a** PBMCs from HBV patients and healthy people were isolated and subjected to RNA extraction, and Q-PCR was performed to evaluate CBFβ mRNA levels. **b** HepG2 cells were treated with serum from HBV patients or healthy people for 8 h. RNA was then extracted, and Q-PCR was performed to evaluate CBFβ mRNA levels. **c** HepG2 cells were treated as indicated. Whole-cell lysates were prepared and subjected to immunoblotting using CBFβ and GAPDH antibodies. RNA was then extracted, and Q-PCR was performed to evaluate CBFβ mRNA levels. **d** HepG2 cells were treated with IFN-λ or HBsAg or both as indicated. Whole-cell lysates were prepared and subjected to immunoblotting as in **c**. **e** HepG2 cells were treated with IL-10 or together with HBsAg as indicated and analyzed as in **c**. All of the data are from three pooled independent experiments and are shown as the mean ± s.d. **p < 0.01, ***p < 0.001. **f** HepG2 cells were transfected with pHBV1.3 plasmids and were treated with HBsAg or HBsAg antibody; 48 h later, HBV DNA or HBeAg in the supernatant was analyzed by Q-PCR or ELISA. All of the data are from three pooled independent experiments and are shown as the mean ± s.d. *p < 0.05, **p < 0.01, ^#^*p* > 0.05. **g** Model depicting the cross talk between type-III interferon and HBV through CBFβ

The above results have already demonstrated that CBFβ was induced after HBV infection and inhibited HBV replication. However, the expression of CBFβ in HBV patients is still unknown. Therefore, PBMCs and serum were isolated from healthy persons and HBV patients, and Q-PCR was performed to evaluate CBFβ expression. The results showed that CBFβ was significantly inhibited in HBV patients compared to healthy persons (Fig. 8a). This finding was consistent with the data shown in Fig. 1b, which showed the suppression of CBFβ in the later time points. Furthermore, HepG2 cells treated with HBV serum showed lower expression of CBFβ than healthy serum-treated cells (Fig. 8b), indicating that HBV-induced cytokines or HBV proteins in the serum might result in the inhibition of CBFβ expression. We previously reported that HBsAg reduced Apobec3G expression,^15^ and hence, we investigated whether HBsAg could also inhibit CBFβ expression. As expected, HBsAg and HepG2.2.15 supernatant treatment inhibited CBFβ expression, and preincubation of the cells with HBsAg antibody before treatment of the cells with HepG2.2.15 supernatant rescued the CBFβ expression (Fig. 8c). HBsAg inhibited IFN-λ-induced CBFβ upregulation, and the basal level of CBFβ was inhibited by HBsAg (Fig. 8d). In addition, CBFβ induction by IL-10 was also inhibited by HBsAg (Fig. 8e). Consistently, HBsAg treatment significantly inhibited the IFN-λ-mediated inhibition of HBV, and HBsAg antibody treatment facilitated the inhibition of HBV replication (Fig. 8f). Taken together, these data suggested an important role of HBsAg in inhibiting CBFβ induction and facilitating HBV replication in HBV patients.

## Discussion

Previous studies have reported that type-III but not type-I IFN is induced after HBV infection. However, it is important to determine the underlying mechanism involved in the cross talk between type-III IFN and HBV, which might explain why some patients are able to eliminate HBV after infection by immune-mediated clearance. We hypothesized that type-III IFN plays an indispensable role in this process, and we found that CBFβ was an inducible gene of type-III IFN but not type-I or type-II IFN and that IL-10 was crucial in the induction of CBFβ by type-III IFN. CBFβ was upregulated after HBV infection and played an inhibitory role in HBV replication. HBx interacted with CBFβ and stabilized CBFβ; this interaction might block the activity of HBx in promoting HBV replication by inhibiting the formation of DDB1–HBx–Smc complexes, while HBsAg might be responsible for the low expression of CBFβ in HBV patients.

IFN-α has been used as a treatment strategy for chronic hepatitis B (CHB) for over 40 years. Although it was slightly improved with combination treatments, such as entecavir (ETV) or tenofovir, IFN-α has not been widely used in the clinic due to its side effects and low efficiency.^34^ Recently, IFN-λ combined with ETV was evaluated in a clinical trial to assess the efficacy of peg-IFN-λ in chronic hepatitis B (CHB) patients.^34,35^ The combination significantly enhanced the treatment efficiency compared to ETV only.^35^ There have been only a few reports that showed the mechanism involved in the inhibition of HBV replication by IFN-λ. Type-III IFN and type-I IFN were reported to share the same JAK/STAT-signaling pathway, leading to the expression of a similar set of genes.^36^ Therefore, in response to viral infection, IFN-λ showed similar antiviral abilities as type-I IFN for the inhibition of the replication of several viruses, including HBV and HCV among others.^37,38^ However, IFN-λ binds to a distinct receptor complex consisting of IL-28R1 (IFN-λR1) and IL-10Rβ (IFN-λR2).^18,39^ Our study showed that CBFβ was specifically induced by IFN-λ1, but not IFN-α or IFN-γ, indicating that the induction might not be dependent on the classical STAT pathway as STAT can be activated by both IFN-λ and IFN-α. Hence, it is of interest to determine the underlying mechanism involved in the induction of CBFβ by IFN-λ. It has been reported that IFN-λ recognizes IL-10Rβ as a subunit of IL-28Rα.^18^ These results were consistent with our study findings that showed that both IFN-λ and IL-10 induced CBFβ production, and we found that IL-10 was critical in IFN-λ-induced CBFβ production. Interestingly, IFN-λ but not IFN-α induces IL-10 in HepG2 cells, which might explain why CBFβ was induced by IFN-λ but not IFN-α.

Consistent with our findings, there are several reports showing that HBV infection can activate the innate immunity of hepatocytes and trigger the production of type-III IFN.^19,40^ However, it has also been reported that both type-I and type-III IFNs are not induced in response to HBV infection.^41^ In our study, we found that IFN-λ, IL-10, and CBFβ were induced at a very early stage after HBV infection, and a prolonged low expression of these proteins was found at the later stage in pHBV1.3-transfected cells or HBV patients. Activation of IFN is the earliest transcriptional response to viral infection; the IFN-λ–IL-10–CBFβ axis might act as a quick response in fighting against HBV in the early stage, and when HBV is uncontrolled in the later stages (e.g., in HBV patients), IFN-λ signaling is inhibited. This hypothesis is consistent with our findings, which showed that CBFβ expression was inhibited in HBV patients and that type-III IFN was not induced in the later stages of HBV infection. Nevertheless, because of the complicated process of the HBV life cycle, the interaction between HBV and IFN signaling needs to be further clarified.

In response to HBV infection, IFN signaling was impaired by HBx through the induction of cytokine signaling 3 and protein phosphatase 2 A suppression.^42^ HBx has been reported to promote Apobec3B degradation by elevating MSL2 expression, resulting in the cccDNA accumulation and hepatocarcinogenesis.^43^ These reports demonstrated the importance of HBx in IFN-signaling disruption and in both interferon and ISG production. Surprisingly, we found that HBx was hijacked by CBFβ, increasing its stability and allowing it to enhance the inhibition of HBV replication. Therefore, our study showed that the host protein CBFβ was stabilized by HBx. More importantly, this stabilization was in favor of host antivirus activity. Additionally, the interaction between CBFβ and HBx might block the transcriptional activity of HBx in promoting HBV replication. However, there was still significant CBFβ-induced suppression of HBV replication in pHBV1.2ΔX-transfected HepG2 cells; CBFβ is the beta subunit of a heterodimeric core-binding transcription factor, suggesting that there might be a HBx-independent mechanism for CBFβ suppression of HBV replication by affecting some other HBV inhibitors.^44^ In addition, we found that HBsAg inhibited IFN-λ-induced CBFβ, which might explain the low expression of CBFβ in HBV patients. Our data suggested that HBx might play a role in the early stage of HBV infection, and HBsAg accumulation resulted in the low expression of CBFβ in the late stages. Our study indicated that there is cross talk between the host and HBV through the HBx–CBFβ–HBsAg axis.

Based on our findings, we proposed a working model for CBFβ in mediating the cross talk between type-III IFN and HBV infection (Fig. 8g). Collectively, we found that CBFβ was induced after HBV infection by type-III IFN in the early stage in an IL-10-dependent manner and that HBx stabilized CBFβ by a protein–protein interaction (PPI). This interaction might block the activity of HBx in promoting HBV replication by inhibiting the formation of DDB1–HBx–Smc complexes, and inversely, the HBV protein HBsAg inhibited CBFβ expression, resulting in low expression of CBFβ in HBV patients. Our study partly explained how type-III IFN inhibited HBV replication and showed that HBx–CBFβ–HBsAg axis may be a new target for the treatment of HBV infection.

## Electronic supplementary material

Table S1

Table S2

Table S3

Figure S1

Figure S2

Figure S3

Figure S4
