# Supplementary material for: Type III interferon-induced CBFβ inhibits HBV replication by hijacking HBx
Source: Cell Mol Immunol. 2018 Mar 9;16(4):357–66. doi: 10.1038/s41423-018-0006-2 (PMC6461963; doi:10.1038/s41423-018-0006-2)
Supplement: Supplementary file 1 — Table S1 [file 41423_2018_6_MOESM1_ESM.doc]

**Table S1. SgRNAs , Q-PCR and cloning primers**

| **GENE** | **FORWARD** | **REVERSR** |
| --- | --- | --- |
| GAPDH | CGGATTTGGTCGTATTGGG | TCTCGCTCCTGGAAGATGG |
| HBV DNA | GAGTGTGGATTCGCACTCC | GAGGCGAGGGAGTTCTTCT |
| pgRNA | TCTTGCCTTACTTTTGGAAG | AGTTCTTCTTCTAGGGGACC |
| IL-10 | GGCACCCAGTCTGAGAACAG | ACTCTGCTGAAGGCATCTCG |
| IL-27 | GAGCTCGTCTTATCTCGGGC | CCCTGATGCCAAGACTCCAG |
| IFNA1 | GCCTCGCCCTTTGCTTTACT | GGATCAGCTCATGGAGGACAGA |
| CBFb | AGTTTGATGAGGAGCGAGCC | TCTTCTTGCCTCCATTTCCTCC |
| IFIT2 | AAGCACCTCAAAGGGCAAAAC | TCGGCCCATGTGATAGTAGAC |
| IFNL1 | CGCCTTGGAAGAGTCACTCA | GAAGCCTCAGGTCCCAATTC |
| IFNL2 and IFNL3 | AGTTCCGGGCCTGTATCCAG | GAGCCGGTACAGCCAATGGT |
| IFNA4  IFNB1  IFNG | ACCTGGTTCAACATGGAAATG  ATGACCAACAAGTGTCTCCTCC  ACTGACTTGAATGTCCAACGCA | ACCAAGCTTCTTCACACTGCT  GCTCATGGAAAGAGCTGTAGTG  ATCTGACTCCTTTTTCGCTTCC |
| CBFβ sgRNA1 | CACCCTCTGGTCGGGCACGACGCG | AAACCGCGTCGTGCCCGACCAGAG |
| CBFβ sgRNA2 | CACCGAGAAGCAAGTTCGAGAACG | AAACCGTTCTCGAACTTGCTTCTC |
| GST-CBFb cloning | TCGCGGCCGCTCTAGAATGCCGCGCGTCGTG | AGGCGCCTGGTCTAGACTAGGGTCTTGTTGTC |
| GST-CBFb（69-90D） | GCCTGCTTC AAACTGGAGAGACAG | CTCCAGTTT GAAGCAGGCAAGGTA |
| GST-CBFb（129-140D） | GAGTTTGAT CAGGCCTTTGAAGAG | AAAGGCCTG ATCAAACTCCAGACA |
