# Supplementary material for: Type III interferon-induced CBFβ inhibits HBV replication by hijacking HBx
Source: Cell Mol Immunol. 2018 Mar 9;16(4):357–66. doi: 10.1038/s41423-018-0006-2 (PMC6461963; doi:10.1038/s41423-018-0006-2)
Supplement: Supplementary file 2 — Table S2 [file 41423_2018_6_MOESM2_ESM.doc]

**Table S2. HBV patients and Healthy Control**

|  | **HCC patients** | | | | **Healthy Control** | |
| --- | --- | --- | --- | --- | --- | --- |
|  | Sex | Age | HBVDNA  (10^6^copies/ml) | ALT | Sex | Age |
| 1 | M | 25 | 116 | 907 | M | 35 |
| 2 | M | 27 | 165 | 1245 | F | 46 |
| 3 | F | 31 | 47.4 | 694 | F | 33 |
| 4 | M | 30 | 4.92 | 280 | M | 28 |
| 5 | F | 28 | 0.047 | 63 | F | 36 |
| 6 | M | 27 | 0.0874 | 1972 | M | 32 |
| 7 | F | 38 | 0.0369 | 441 | M | 39 |
| 8 | M | 40 | 5.77 | 499 | F | 58 |
| 9 | M | 48 | 0.27 | 706 | M | 55 |
| 10 | F | 41 | 137 | 356 | F | 58 |
| 11 | M | 38 | 49.5 | 272 | F | 50 |
| 12 | M | 27 | 113 | 1105 | F | 33 |
| 13 | F | 47 | 346 | 60 | M | 37 |
| 14 | F | 50 | 154 | 85 | M | 29 |
| 15 | F | 36 | 2.32 | 930 | M | 33 |
| 16 | F | 29 | 5.58 | 230 |  |  |
| 17 | M | 24 | 52.7 | 280.8 |  |  |
| 18 | M | 53 | 23.5 | 87.2 |  |  |
| 19 | F | 56 | 21.6 | 126.7 |  |  |
| 20 | M | 35 | 102 | 112.8 |  |  |
| 21 | M | 25 | 7.55 | 1316 |  |  |
| 22 | M | 43 | 2.83 | 293 |  |  |
| 23 | M | 30 | 2.7 | 293 |  |  |
| 24 | M | 42 | 9.07 | 138 |  |  |
| 25 | F | 47 | 3.46 | 60 |  |  |
| 26 | M | 36 | 4.54 | 66 |  |  |
| 27 | F | 50 | 1.54 | 85 |  |  |
| 28 | M | 25 | 6.62 | 366 |  |  |
