# Supplementary material for: Type III interferon-induced CBFβ inhibits HBV replication by hijacking HBx
Source: Cell Mol Immunol. 2018 Mar 9;16(4):357–66. doi: 10.1038/s41423-018-0006-2 (PMC6461963; doi:10.1038/s41423-018-0006-2)
Supplement: Supplementary file 3 — Table S3 [file 41423_2018_6_MOESM3_ESM.doc]

**Table S3. HBV infected patients with IFN treatment**

**before treatment 17 weeks after treatment**

| NO | AST0 | ALT0 | HBEAG0 | HBV0 | AST17 | ALT17 | HBEA G17 | HBV17 |
| --- | --- | --- | --- | --- | --- | --- | --- | --- |
| 1 | 188 | 280 | 963.762 | 170000000 | 63 | 64 | 13.754 | 119000 |
| 2 | 60 | 106 | 861.489 | 170000000 | 59 | 96 | 782.74 | 170000000 |
| 3 | 130 | 162 | 903.773 | 124000000 | 104 | 141 | 657.992 | 14500000 |
| 5 | 78 | 152 | 1368.318 | 170000000 | 21 | 17 | 35.267 | 165000 |
| 6 | 111 | 293 | 1240 | 170000000 | 55 | 117 | 667.605 | 170000000 |
| 7 | 95 | 219 | 675.52 | 170000000 | 73 | 135 | 434.09 | 170000000 |
| 8 | 54 | 108 | 0.455 | 2560000 | 44 | 65 | 0.349 | 296 |
| 9 | 89 | 145 | 1028.726 | 170000000 | 80 | 82 | 20.289 | 1310000 |
| 10 | 89 | 91 | 0.433 | 25400000 | 193 | 273 | 0.415 | 224 |
