# Supplementary figures and images for: Type III interferon-induced CBFβ inhibits HBV replication by hijacking HBx
Source: Cell Mol Immunol. 2018 Mar 9;16(4):357–66. doi: 10.1038/s41423-018-0006-2 (PMC6461963; doi:10.1038/s41423-018-0006-2)

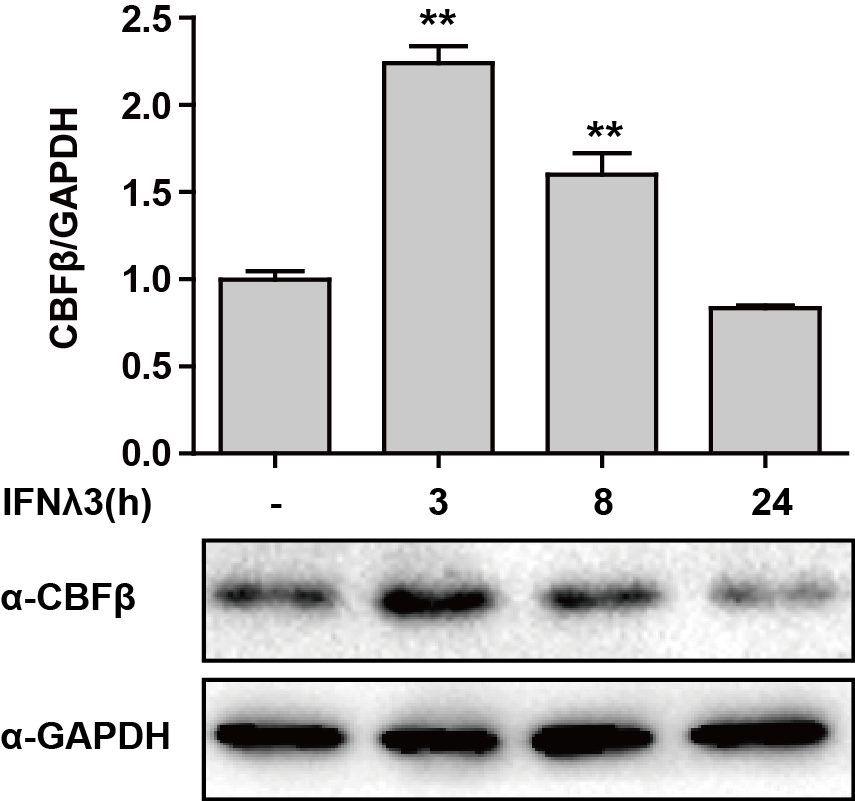

Supplement: Supplementary file 4 — Figure S1 [file 41423_2018_6_MOESM4_ESM.jpg]

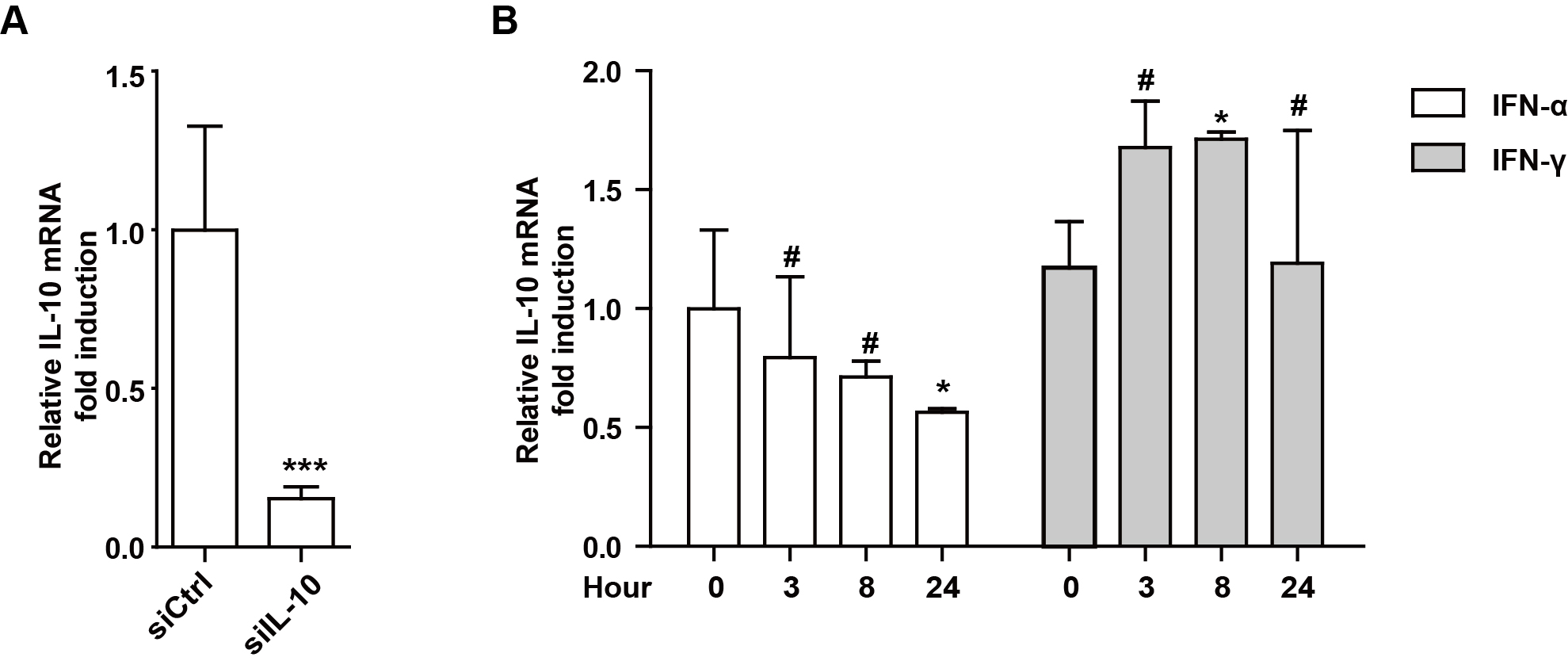

Supplement: Supplementary file 5 — Figure S2 [file 41423_2018_6_MOESM5_ESM.jpg]

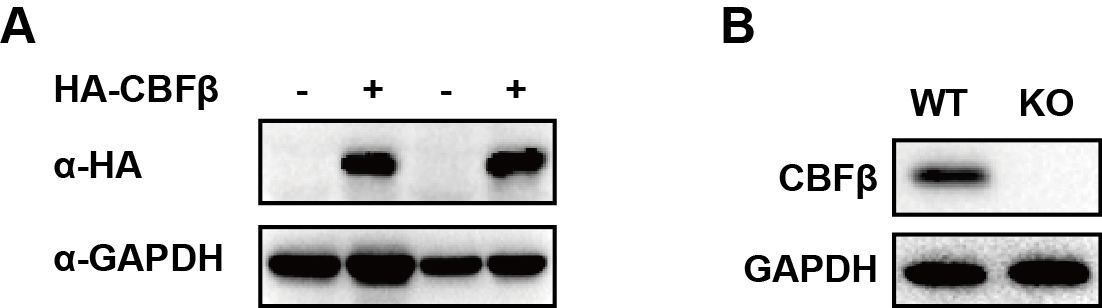

Supplement: Supplementary file 6 — Figure S3 [file 41423_2018_6_MOESM6_ESM.jpg]

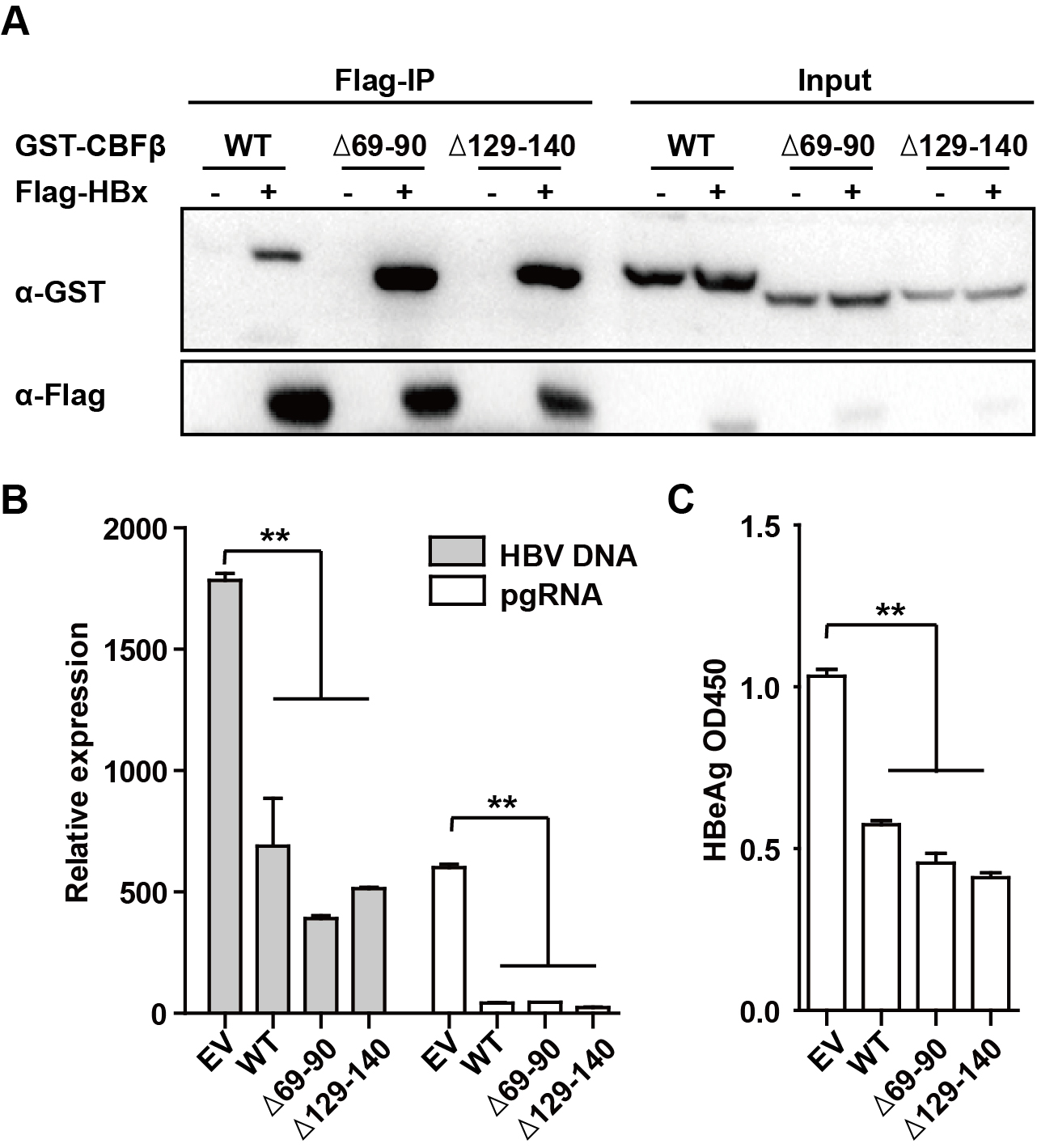

Supplement: Supplementary file 7 — Figure S4 [file 41423_2018_6_MOESM7_ESM.jpg]
